# Nursing students’ perceptions on clinical learning environment and mental health: a multicenter study

**DOI:** 10.1590/1518-8345.5577.3528

**Published:** 2022-07-08

**Authors:** Gul Sahin Karaduman, Gizem Kubat Bakir, Maria Margarida Santana Fialho Sim-Sim, Tulay Basak, Sonay Goktas, Aelita Skarbalienė, Indrė Brasaitė-Abromė, Manuel José Lopes

**Affiliations:** 1 University of Health Sciences Turkey, Gulhane Training and Research Hospital, Ankara, Turquia.; 2 Maltepe University, School of Nursing, İstanbul, Turquia.; 3 University of Évora, Comprehensive Health Research Centre (CHRC), Évora, Portugal.; 4 University of Health Sciences Turkey, Gulhane Faculty of Nursing, Ankara, Turquia.; 5 University of Health Sciences Turkey, Hamidiye Faculty of Nursing, İstanbul, Turquia.; 6 Klaipeda University, Faculty of Health Science, Department of Nursing, Klaipeda, Lituânia.

**Keywords:** Education, Students, Clinical, Learning, Environment, Mental Health, Educação, Estudantes, Clínica, Aprendizagem, Meio Ambiente, Saúde Mental, Educación, Estudiantes, Clínica, Aprendizaje, Ambiente, Salud Mental

## Abstract

**Objective:**

this study aimed at evaluating the perceptions of Nursing students from public universities in three European Union countries on mental health and clinical learning environments, a topic that has been rarely investigated in the literature.

**Method:**

data collection took place using a demographic data form, the Clinical Learning Environment, Supervision and Nurse Teacher Scale, and the Mental Health Continuum Short Form. A total of 571 participants from Turkey, Lithuania and Portugal were included in the study.

**Results:**

there was a significant difference among the three groups regarding clinical learning environment and mental health status (p<.001). Supervision was the most valued element. The Portuguese students presented the highest mean in the Mental Health Continuum Short Form and Clinical Learning Environment, Supervision and Nurse Teacher Scale scores (p<.001). Age, gender and mental health were effective in the Clinical Learning Environment, Supervision and Nurse Teacher Scale scores.

**Conclusion:**

the results indicated that the Mental Health Continuum Short Form and Clinical Learning Environment, Supervision and Nurse Teacher Scale scores obtained by the Portuguese Nursing students were higher. It was also revealed that the students’ perceptions on the clinical learning environment were affected by age and gender, and that their perceptions on mental health were influenced by the Clinical Learning Environment, Supervision and Nurse Teacher scale scores.

## Resumo

**Objetivo:**

este estudo teve como objetivo avaliar as percepções dos estudantes de Enfermagem das universidades públicas de três países da União Europeia sobre saúde mental e ambientes de aprendizagem clínica, tema pouco investigado na literatura.

**Método:**

a coleta de dados ocorreu por meio de um formulário de dados demográficos, a Escala Ambiente de Aprendizagem Clínica, Supervisão e Professor de Enfermagem e o *Mental Health Continuum Short Form*. Um total de 571 participantes da Turquia, Lituânia e Portugal foram incluídos no estudo.

**Resultados:**

houve uma diferença significativa entre os três grupos em relação ao ambiente de aprendizagem clínica e estado de saúde mental (p <0,001). A supervisão foi o elemento mais valorizado. Os estudantes portugueses apresentaram a média mais elevada nos escores do *Mental Health Continuum Short Form* e Ambiente de Aprendizagem Clínica, Supervisão e Professor de Enfermagem (p<0,001). Idade, sexo e saúde mental influíram nos escores do Ambiente de Aprendizagem Clínico, Supervisão e Professor de Enfermagem.

**Conclusão:**

os resultados indicaram que os escores do *Mental Health Continuum Short Form* e Ambiente de Aprendizagem Clínica, Supervisão e Professor de Enfermagem obtidos pelos estudantes de Enfermagem portugueses foram mais elevados. Revelou-se também que as percepções dos alunos sobre o ambiente de aprendizagem clínica foram afetadas pela idade e sexo, e que suas percepções sobre saúde mental foram influenciadas pelos escores da escala Ambiente de Aprendizagem Clínica, Supervisão e Professor de Enfermagem.

## Resumen

**Objetivo:**

este estudio tuvo como objetivo evaluar las percepciones de estudiantes de enfermería de universidades públicas de tres países de la Unión Europea sobre la salud mental y los Ambientes de Aprendizaje Clínico, tema poco estudiado en la literatura.

**Método:**

la recolección de datos se realizó mediante un formulario de datos demográficos, la Escala de Evaluación de Ambiente de Aprendizaje Clínico, Supervisión y Profesor de Enfermería y el *Mental Health Continuum Short Form*. Se incluyeron en el estudio un total de 571 participantes de Turquía, Lituania y Portugal.

**Resultados:**

hubo una diferencia significativa entre los tres grupos con respecto al Ambiente de Aprendizaje Clínico y al estado de salud mental (p < 0,001). La supervisión fue el elemento más valorado. Los estudiantes portugueses obtuvieron los puntajes promedio más altos en el *Mental Health Continuum Short Form* y Ambiente de Aprendizaje Clínico, Supervisión y Profesor de Enfermería (p<0,001). La edad, el sexo y la salud mental influyeron en los puntajes de Ambiente de Aprendizaje Clínico, Supervisión y Profesor de Enfermería.

**Conclusión:**

los resultados indicaron que los estudiantes de Enfermería portugueses obtuvieron los puntajes más altos en el *Mental Health Continuum Short Form* y Ambiente de Aprendizaje Clínico, Supervisión y Profesor de Enfermería. También se reveló que las percepciones de los estudiantes sobre el Ambiente de Aprendizaje Clínico se vieron afectadas por la edad y el género, y que sus percepciones sobre la salud mental fueron influenciadas por los puntajes de la escala de evaluación de Ambiente de Aprendizaje Clínico, Supervisión y Profesor de Enfermería.

**Highlights:**

(1) CLES-T scores of Portuguese nursing students were higher than Turkish, Lithuanian.

(2) The clinical learning environment was affected by age, gender, and mental health.

(3) The mental health was affected by the learning environment, supervisor, professor.

## Introduction

The clinical practice is a vital component of Nursing education, aiding Nursing students in using their cognitive, psychomotor and affective skills in combination^1^ ^-^ ^2^. Nursing students participate in a new clinical environment mainly to gain practical experience. They may feel anxious and uncertain when first exposed to the complex healthcare environment^3^. They may also face situations that require coping skills such as systematic and dynamic clinical environments, a process of getting used to these environments, anxiety caused by lack of knowledge or skills, fear of making mistakes, and caring for different severely-ill patients^4^. A number of studies have shown that the students may experience stress due to several factors such as absence of theoretical training, lack of skills in clinical practices to assume responsibility for the patients, time pressure, lack of motivation and accommodation, social life, new responsibilities, and adaptation to a new environment^5^ ^-^ ^6^. These factors make them feel vulnerable, and receiving insufficient support can be detrimental to their learning^3^.

Stress affects Nursing students’ behavior in hospital wards as well. Moreover, it also exerts a significant impact on their mental health and well-being^4^ ^,^ ^7^. Mental health represents a combination of emotional, psychological, and social well-being and certain humanitarian harmony necessary for an individual to be mentally healthy^8^. Assessing and promoting Nursing students’ mental health is seen as an important requirement that promotes health and strengthens the curriculum^9^. Mental health and clinical learning environments are important factors for Nursing students to acquire skills for professional roles and these environments allow them to provide care in a controlled process. In this way, the students learn clinical procedures and also develop their practical, problem-solving, decision-making and communication skills that enable them to communicate both with healthy individuals and with patients^10^.

In many parts of the world, undertaking a clinical practice is an essential component of nurses’ education^3^. Therefore, nurse educators need to evaluate whether the clinical environments are suitable for learning and should take necessary measures to facilitate the students’ learning and development^10^. In Nursing education, clinical environments are important to achieve the desired educational quality and, thus, should be designed in such a way to support the students’ social and mental development^5^ ^,^ ^10^.

According to the World Health Organization (WHO)^11^, there is a need “to raise the level of Nursing and Midwifery education in the European Region, to create evidence-based knowledge on these areas”. On this matter, the Bologna Declaration announced some reforms to be achieved, including the establishment of an exhaustive framework of degrees and cycles in an open European area for higher education and a common degree level system for undergraduates, as well as the enhancement and facilitation of students’ and teachers’ commuting, improvements in the recognition of degrees and academic qualifications, and the creation of a European Credit Transfer and Accumulation System (ECTS)^12^. Nursing education has been transformed over the last decades and this transformation is continuing. The European Union and the Bologna Process have influenced changes in Nursing education^13^.

In addition to the Bologna process, Nursing schools acknowledge the importance of exposing students to diverse cultures through international exchanges and study-abroad experiences that enrich knowledge, understanding and the healthcare practice^13^. Exchange programs such as Erasmus Exchange provide students with the opportunity to recognize professional self-confidence development, different Nursing education systems, healthcare services and different cultures. Nursing educators need to socialize their students into the cultural context of the Nursing practice system. Addressing the advantages, disadvantages and benefits of dealing with a diverse group of students leads to a profound effect on the prospects of the Nursing practice healthcare environment^14^.

Each country has its own legislation, culture, health needs, health philosophies and structures, and economic situations. Especially, the education systems differ across the countries. Portugal has a four-year polytechnic education system, while Turkey and Lithuania offer four-year University education. Nurses’ practical training takes place in a real Nursing environment, in a hospital or in other personal healthcare institutions, where the competencies of future nurses are developed. Considering the unemployment rate in terms of the Nursing profession, the lowest is seen in Lithuania and the highest, in Turkey. The nurses’ *per capita* income level presents its highest value in Lithuania and the lowest in Turkey. This leads to job migration of nurses to other European countries due to better remuneration, easy access to recruitment networks and, sometimes, previous experience in the Erasmus Exchange program^15^ ^-^ ^17^. 

Various studies have been conducted to evaluate clinical learning environment and mental health separately^5^ ^-^ ^7^ ^,^ ^9^ ^-^ ^10^. However, an extensive literature review indicated no multicultural studies comparing clinical environment and mental health in Nursing students. International collaborative research between countries in the field of Nursing education raises the profile of Nursing as a postgraduate profession. Given the cultural differences among the three countries, we considered that Nursing students’ perceptions on clinical learning environments and mental health could differ as well. We also considered that the findings to be obtained from this study would identify the Nursing students’ attitudes and help them enhance their intercultural understanding when applying the health practices implemented in other cultures. To that end, this study aimed at evaluating the perceptions of Nursing students from public universities in three European Union countries on mental health and clinical learning environments, a topic that has been rarely investigated in the literature. 

To this end, the following research questions were addressed:

- Which are the Nursing students’ perceptions on clinical learning environments?
- Which are the Nursing students’ perceptions on the elements of clinical learning environments (supervision, teacher, environment)? Which of these elements is most valued in each country?
- Which are the Nursing students’ perceptions on mental health?
- Which is the relationship of the clinical learning environment with mental health and the demographic characteristics?

## Method

### Study design and locus

This is a cross-sectional, multicenter and descriptive study guided by the STROBE (Strengthening the Reporting of Observational Studies in Epidemiology) tool^18^, which analyzed the perceptions of Nursing students from three different European Union countries on mental health and clinical learning environments. The study was conducted in Turkey, Portugal and Lithuania between May 2019 and February 2020. 

### Sample definition

All the participants were undergraduate Nursing students from provinces of Turkey, Portugal and Lithuania, accounting for a total of 500, 360 and 340 students, respectively. Sample size for each school was calculated using an online software calculator^19^. At a 95% confidence level of and a 0.5 confidence interval, optimal sample size was calculated at 217, 186, and 181 for the Turkish, Portuguese and Lithuanian students, respectively. However, 10 students from Turkey and 3 from Lithuania were excluded from the study as they provided incomplete or incorrectly completed questionnaire forms. As a result, a total of 207 students from Turkey, 186 from Portugal and 178 from Lithuania were included in the study.

### Participants

The Nursing students eligible to participate in the study included those who had completed at least one internship program, were national or native speakers of the study country and were aged at least 18 years old. Nursing students from a different country, such as from the Erasmus exchange program, did not participate in the study. All the students who volunteered to participate in the study filled in the data collection form between May 2019 and February 2020. In the Lithuanian school, the data were collected from second-, third- and fourth-year students, as there was no internship program for first-year students. Similarly, in the Turkish school, the data were collected from first-, second- and third-year students, as no new students were enrolled in the school in the year 2016. In the Portuguese school, the data were obtained from first-, second-, third- and fourth-year students. The sample consisted of 207 students from Turkey, 186 from Portugal and 178 from Lithuania. The participants were not allocated through randomization. The researchers were aware of the participants’ demographic characteristics. The data analyst was not aware of the participants’ demographic characteristics.

### Ethical aspects

The study was conducted in accordance with the principles expressed in the Declaration of Helsinki. Approval was obtained from the ethics committees and each institution prior to initiation of the study (University of Évora, São João de Deus School of Nursing; Approval No: GD/16331/2019, Date: 05.03.2019; University of Health Sciences from Turkey, Gülhane Nursing School; Approval No: 19/294; Date: 10.12.2019; Klaipeda University School of Health Sciences; Approval No: 46Sv-SL-6, Date: 26.10.2019). Participation was on a voluntary basis and all participants had the right to accept or refuse participation. A written informed consent was obtained from each participant and all were informed that their credentials would be kept confidential, and that they would not be under any financial burden.

### Instruments used to collect the information

The data collection forms included a demographic data form, the Clinical Learning Environment, Supervision and Nurse Teacher Scale (CLES+T)^20^ and the Mental Health Continuum Short Form (MHC-SF)^21^.

### Demographic data form

The form consisted of six questions probing the students’ demographic characteristics and daily habits (gender, age, course year, place of residence, home-school commute means of transportation, and scholarship).

### Clinical Learning Environment, Supervision and Nurse Teacher Scale (CLES+T)

CLES+T was developed by Saarikoski and Leino-Kilpi in 2002 and was revised in 2008^22^ ^-^ ^23^. It consists of 34 items with five subdimensions: Pedagogical atmosphere (9 items), Leadership style of the ward manager (4 items), Premises of Nursing in the ward (4 items), Supervisory relationship (8 items), and Role of the nurse teacher (9 items). All items are rated based on a 5-point Likert scale (1=fully disagree, 2=disagree to some extent, 3=neither agree nor disagree, 4=agree to some extent, and 5=fully agree)^20^ ^,^ ^22^ ^-^ ^23^. A different version of CLES+T was administered for each country (Portuguese version^24^, Turkish version^25^ and Lithuanian version^26^). In the original study^20^, reliability of CLES+T was shown with a Cronbach’s alpha value ranging from .77 to .96 for all the items. As for the other versions, the Cronbach’s alpha values varied from .70 to .97 for the Portuguese version, from .76 to .90 for the Turkish version and from .85 to .95 for the Lithuanian version. In our study, the Cronbach’s alpha value was .950 for the Portuguese version, .947 for the Turkish version, and .966 for the Lithuanian version of CLES+T, and the Cronbach’s alpha value for the total sample was .961.

### Mental Health Continuum Short Form (MHC-SF)

MHC-SF was developed to evaluate mental state from a continuum perspective^27^. It has 14 items, all worded positively, and consists of a 5-point Likert scale (0, never; 1, once or twice a month; 2, about once or twice a week; 3, two or three times a week; 4, almost every day; 5, every day). The total score is calculated based on the sums of all items and higher scores indicate better mental health. The Cronbach’s alpha value of the original scale was reported as .89^21^.

The Portuguese version of MHC-SF was developed^28^. The Cronbach’s alpha value of this version was reported to be .93^28^. The Turkish version of MHC-SF was developed and its Cronbach’s alpha value was reported as .90^29^. The Lithuanian version of MHC-SF was developed and its Cronbach’s alpha value was reported as .91^30^. In our study, the Cronbach’s alpha value was .846 for the Portuguese version, .946 for the Turkish version, and .966 for the Lithuanian version of MHC-SF. Moreover, the Cronbach’s alpha value for the total sample was .937.

### Data treatment

The study data were collected from volunteer students who were involved in at least one clinical practice between May 2019 and February 2020, after obtaining approvals from the universities and ethical committee permissions. The data were collected by the researchers from each country, after explanation of the research objectives and data confidentiality. The students who were available and had interest in participating signed the Informed Consent Form. The data were collected while the students were at the school, not in the clinical practice. Data collection lasted approximately 10 minutes. The number of days that the students are in the school decreases as their course year increases. Therefore, data were collected in a shorter time in the first year of the course than in the fourth. This period ranged from two to five weeks for each year of the students. It took a total of 10 months to collect the data from all countries. A pilot study was conducted to test the validity and reliability of the instruments used, and 25 participants from each country participated in this pilot study. It yielded a Cronbach’s alpha value above .70 for each instrument. The participants received no financial incentive to participate in the study. 

### Data analysis

The data were analyzed using IBM SPSS^®^ (IBM Corp., Armonk, NY, USA), version 24.00. The dependent variables of the research were the CLES+T and mental health scores and the independent variables were demographic data such as country, age, gender and Nursing course year. The descriptive variables were expressed as frequencies (n), percentages (%), mean values, and standard deviations (SDs). Normal data distribution was assessed by means of the Kolmogorov-Smirnov test. The reliability analysis was performed by calculating Cronbach’s alpha coefficients. The three groups were compared using One-way ANOVA followed by Tukey’s HSD test. Paired *t*-test was used to compare the mean values of two dependent variables. Construct validity, both for the entire sample and by each country’s sample, was assessed using Principal Component Factor Analysis (PCFA). Multiple Linear Regression (MLR) tests were applied to identify the predictors of each of the elements of the learning process (environment, supervision, teacher). The predictive outcomes were scholarship, age, gender and mental health, and the outcome variables were learning environment, supervision and teacher. A *p-value*<.05 was accepted as significant.

## Results

### Characteristics of the sample

The study sample consisted of 571 Nursing students. A significant difference was found among the three countries with regard to age (F_(2.563)_=51.802; *p*<.001), whereby the Turkish students were significantly younger than those from the other countries and the Portuguese students were significantly younger than the Lithuanian (*p*<.005). The sociodemographic and educational characteristics of the students are presented in Table 1.

**Table 1:** Sociodemographic characteristics and daily habits of the Portuguese, Turkish and Lithuanian students (n=571). Évora-Portugal, Ankara-Turkey, Klaipeda-Lithuania, 2019-2020

Variables		Portuguese	Turkish	Lithuanian	Total
		Mean±SD^*^	Mean±SD^*^	Mean±SD^*^	Mean±SD^*^
Age		22.01±2.72	20.26±2.20	23.83±4.93	21.93±3.70
		n (%)	n (%)	n (%)	n (%)
**Gender**	Male	29 (15.6)	34 (16.4)	3 (1.7)	66 (11.6)
	Female	157 (84.4)	173 (83.6)	175 (98.3)	505 (88.4)
**Nursing course year**	1^st^	19 (10.2)	52 (25.1)	-	71 (12.4)
	2^nd^	58 (31.2)	47 (22.7)	47 (26.4)	152 (26.6)
	3^rd^	52 (28)	108 (52.2)	58 (32.6)	218 (38.2)
	4^th^	57 (30.6)	-	74 (41)	130 (22.8)
**Place of residence**	Family home	66 (35.5)	75 (36.2)	43 (24.2)	184 (32.2)
	Student housing	26 (14.0)	86 (41.5)	24 (13.5)	136 (23.8)
	Rented room	46 (24.7)	1 (.5)	48 (27.0)	47 (8.2)
	House with peers	38 (20.4)	32 (15.5)	-	118 (20.7)
	Other	10 (5.4)	13 (6.3)	63 (35.4)	86 (15.1)
**Home-school commute**	On foot	114 (61.3)	75 (36.2)	25 (14.0)	214 (37.5)
	Bus	9 (4.8)	122 (58.9)	43 (24.2)	174 (30.5)
	Own car	52 (28)	2 (1.0)	108 (60.7)	162 (28.4)
	Car driven by other person	7 (3.8)	4 (1.9)	2 (1.1)	13 (2.3)
	Train	-	4 (1.9)	-	4 (.7)
	Motorcycle	3 (1.6)	-	-	3 (.5)
	Bicycle	1 (.5)	-	-	1 (.2)
**Scholarship**	Yes	90 (48.4)	99 (47.8)	9 (5.1)	198 (34.7)
	No	96 (51.6)	108 (52.2)	169 (94.9)	373 (65.3)
**Total**		186 (32.6)	207 (36.3)	178 (31.2)	571 (100)

^*^Standard Deviation

### Nursing students’ perceptions on Clinical Learning Environments

The factor solution for the dimensions was similar across the three countries, showing: 1) Supervisory relationship (8 items), 2) Role of the nurse teacher (9 items) and 3) Learning environment (17 items). The explained variance for the total scale and for each country presented its highest value for the Lithuanian sample (66.67%) and the lowest for the Portuguese sample (53.70%). All the factors in the components had an eigenvalue above .40, except for item 26 in the Portuguese sample.

The ANOVA test indicated significant differences among the three groups (F_(2.555)_=65.896; *p*<.001), whereas the mean value for the Portuguese students (M=4.07±.521) was significantly higher than that of the Lithuanian (M=3.71±.794) and Turkish (M=3.28±.698) students.

### Nursing students’ perceptions on the elements of Clinical Learning Environments

The results indicated that the Portuguese students had the highest means in all dimensions, while only some of them established a significant difference with those of other groups. In the *environment* dimension, no significant difference was found between the Portuguese and Lithuanian students (*p*=.090), whereas a significant difference was identified between the students from those two countries and their peers from Turkey (*p*<.001). In the *supervision* dimension, a significant difference was found among all three groups (*p*<.001). As for the *teacher* dimension, no significant difference was found between the Turkish and Lithuanian students (*p*=.972), whereas a significant difference was in fact identified between the students from those two countries and their peers from Portugal (*p*<.001).

The most valued element of the clinical learning environments was *supervision* for the Portuguese and Turkish students, while it was *environment* for the Lithuanian students. In contrast, the least valued element was *teacher* for all three groups. Moreover, a significant difference was found between the Portuguese and Lithuanian students’ perceptions on *teacher*, whereas no significant difference was found with those of the Turkish students (*p*=.088), according to Table 2. 

**Table 2:** Difference between the Portuguese, Turkish and Lithuanian students’ perceptions on Clinical Learning Environment (n=571). Évora-Portugal, Ankara-Turkey, Klaipeda-Lithuania, 2019-2020

Nationality	Paired group	Mean±SD	t	p^*^
**Portugal**	Learning Environment Supervision	4.06±0.53	-3.81	*0.00*
		4.24±0.81		
	Supervision Teacher	4.24±0.81	4.93	*0.00*
		3.92±0.74		
	Learning Environment Teacher	4.06±0.53	2.45	*0.01*
		3.92±0.74		
**Turkey**	Learning Environment Supervision	3.30±0.74	-1.13	0.25
		3.35±0.90		
	Supervision Teacher	3.35±0.90	2.45	*0.01*
		3.20±0.85		
	Learning Environment Teacher	3.30±0.74	1.71	0.08
		3.20±0.85		
**Lithuania**	Learning Environment Supervision	3.91±0.80	0.81	0.41
		3.84±1.04		
	Learning Supervision Teacher	3.84±1.04	8.75	*0.00*
		3.18±1.13		
	Learning Environment Teacher	3.91±0.80	9.68	*0.00*
		3.18±1.13		

^*^Paired *t*-test

### Nursing students’ perceptions on Mental Health

The Portuguese students had the highest MHC-SF mean score (3.74±0.72), followed by their Lithuanian counterparts (3.53±1.02). However, this difference was statistically insignificant (*p*=.069). On the other hand, the Turkish students obtained a mean of 3.05±1.02, which was statistically different from those of the other two groups (*p*<.001).

### Relationship of Clinical Learning Environment with Mental Health and demographic characteristics

The results showed that age (β=.084; CI=From .003 to .033; *p*=.019) and MHC-SF (β=0.55; CI=From 0.38 to 0.49; *p*<0.001) were positively correlated, explaining 31.3% of the variance in the clinical learning environment. In the second model, gender was negatively correlated (β=-0.07; CI=From -0.47 to -0.01; *p*=0.03) and MHC-SF (β=0.45; CI=From 0.38 to 0.53; *p*<0.001) was positively correlated, explaining 20.4% of the variance in the *supervision* dimension. In the third model, gender was also negatively correlated (β=-0.07; CI=From -0.45 to -0.00; *p*=0.04) and MHC-SF (β=0.46; CI=From 0.38 to 0.52; *p*<0.001) was positively correlated, explaining 20.8% of the variance in the *teacher* dimension, according to Table 3.

**Table 3:** Regression analysis of the Clinical Learning Environment subdimensions with Mental Health and demographic characteristics (n=571). Évora-Portugal, Ankara-Turkey, Klaipeda-Lithuania, 2019-2020

Predictive Variables	Learning Environment					Supervision					Teacher				
	B^*^	Sig.	Beta	Lower 95%	Upper 95%	B	Sig.	Beta	Lower 95%	Upper 95%	B	Sig.	Beta	Lower 95%	Upper 95%
**Constant^†^**	1.86	0.00		1.46	2.27	2.12	0.00		1.57	2.68	2.41	0.00		1.87	2.96
**Scholarship**	0.06	0.25	0.04	-0.04	0.18	-0.03	0.65	-0.01	-0.19	0.12	-0.05	0.51	-0.02	-0.20	0.10
**Age**	0.01	0.01	0.08	0.00	0.03	0.01	0.09	0.06	-0.00	0.03	-0.01	0.27	-0.04	-0.03	0.00
**Gender**	-0.15	0.06	-0.06	-0.32	0.01	-0.24	0.03	-0.07	-0.47	-0.01	-0.23	0.04	-0.07	-0.45	-0.00
**Mental Health Total Score**	0.43	0.00	0.55	0.38	0.49	0.45	0.00	0.45	0.38	0.53	0.45	0.00	0.46	0.38	0.52
**Model**	R	R Square	Adjusted R Square	Sig. F Change	Durbin-Watson	R	R Square	Adjusted R Square	Sig. F Change	Durbin-Watson	R	R Square	Adjusted R Square	Sig. F Change	Durbin-Watson
	0.56	0.31	0.31	0.00	1.91	0.45	0.21	0.20	0.00	1.91	0.46	0.21	0.20	0.00	1.98

^*^Unstandardized Beta; ^†^Used instead of random variables

## Discussion

The study provided valuable insight for the Nursing students’ perceptions on clinical learning environments and mental health. The results regarding the participants’ demographic characteristics, CLES+T scores and MHC-SF scores were discussed in line with the relevant literature.

### Characteristics of the sample

The mean age of the participants in this study presented its lowest value in Turkey and the highest in Lithuania. This difference could be due to the absence of first-year Nursing students in the Lithuanian group and of fourth-year Nursing students in the Turkish group. 

In terms of gender, the percentage of males was lower among the Lithuanian students than in the other groups. When the studies conducted with Nursing students in Turkey, Portugal and Lithuania were examined, it was verified that the results are similar^31^ ^-^ ^32^. Nursing is one of the most female-dominated professions all over the world. It is thought that a professional group that integrates the characteristics of both genders could contribute positively to the scientific and state-of-the-art development of the profession by diverting attention from gender.

### Nursing students’ perceptions on Clinical Learning Environments

In our study, the *environment*, *supervision* and *teacher* mean scores of the Portuguese students were found to be higher. Clinical learning requires appropriate and sufficient personnel covered by teachers, supervisors and environment^33^. It is thought that this difference in the study results was due to the inadequacy of the application area, to the excess number of students, and to the lack of supervisors and teachers. The difference in the education system can be shown as another reason for these results.

In line with these results, it is necessary to support Nursing students in the clinical experience. Closing the gap between clinical and theoretical education is one of the main purposes in Nursing education. Clinical education is not only a practice carried out with instructors, but also an educational experience that the student should pursue with experienced clinical nurses^34^. 

### Nursing students’ perceptions on the most valued elements in Clinical Learning Environments

The results indicated that the most valued element was *supervision* for the Portuguese and Turkish students while it was *environment* for the Lithuanian students. It is thought that this difference was due to the fact that Nursing students in Portugal and Turkey were unfamiliar with the field of practice and did not have sufficient skills in Nursing interventions; therefore, they expected support from supervisors, especially in applications that require skills.

The clinical environment is the best area in which clinical decision-making can be taught and developed. Supervisors are key individuals who help students bridge the gap between Nursing theory and practice. A good supervisor facilitates the provision of safe and effective care to the patients. The students’ relationship with the supervisor can improve their clinical practice skills^1^. Local developments regarding the concept of “supervisor” continue in all three countries.

### Nursing students’ perceptions on Mental Health

The Portuguese students obtained the highest MHC-SF scores, followed by their Lithuanian and Turkish counterparts, respectively. It is stated that social support exerts a positive effect on mental health and well-being^35^. It is thought that the higher mental health scores obtained by the Portuguese Nursing students could be related to perceived social support factors. The fact that the Portuguese Nursing students have higher *supervision*, *teacher* and *environment* scores than the other groups also support these findings.

Nursing students spend a long time on training in the hospitals, which are stressful environments^36^. Learning in a real clinical environment requires a view that captures the complexity of such setting. Therefore, the supervision, teacher and environment factors in a clinical learning environment should support Nursing students’ mental health. It is imperative to establish positive mental health interventions that will facilitate expansion of a satisfied and healthy population of Nursing students^37^.

### Relationship of Clinical Learning Environment with Mental Health and demographic characteristics

The regression analysis revealed a significant positive correlation between age and clinical learning environment. Age is thought to be directly related to decision-making processes in the clinical practice^38^. Accordingly, as the students’ age increases, their perceptions on their clinical environment are likely to be positively affected. The mean age of all the students participating in the study is similar among them. The age for Nursing training initiation is similar in all three countries. It is thought that, as age increases, the students’ experiences also increase and, accordingly, the anxiety and stress levels decrease.

On the other hand, a significant negative correlation was found between gender and two elements, *supervision* and *teacher,* and it was also revealed that female students’ scores for *supervision* and *teacher* were lower than those of male students. It should be considered that the different learning styles of female and male students may also be the cause for this situation^39^. In addition, the impact of direct interaction with patients in a female-dominated profession is particularly significant for male rather than for female Nursing students. The low number of male students increases their visibility in the clinical learning environment. This causes the patients to question male students’ capabilities when they interact with them to perform clinical practices. This shows that these students are exposed to gender bias and labeling^40^. For this reason, supervisors and teachers should be careful about the problems experienced by male students in clinical learning environments due to gender.

Our results indicated that mental health affects all the elements of the clinical learning environment. The quality of the “learning environment” was a key influence on Nursing students’ emotional well-being. Teacher, supervision and teaching approaches, academic expectations and availability of learning resources are important factors that affected their emotional well-being as well as their academic performance^41^. In line with these results, it is thought that Nursing students should be supported in this regard, considering that being in the hospital can cause stress. In addition, a clinical learning environment with minimal stress, supportive institutional policies and adequate facilities are necessary to help the students meet the learning demands optimistically.

The results of this research contribute to the identification of the clinical learning environment and mental health of Nursing students by means of a multicenter study. It is hoped that the findings presented may contribute to the practice of university teachers and administrators by stimulating discussion about curriculum changes and strategies to improve the students’ satisfaction and success in the clinical learning environment.

### Limitations

This research has some limitations. The study was conducted in Turkish, Portuguese and Lithuanian Nursing schools; thus, its results can only be generalized to the three countries’ Nursing schools. Another study limitation was that not all the Nursing students attending all classes could be reached in Turkey and Lithuania. The Portuguese school had students from all classes; the Lithuanian school did not have any internship program for first-year students; and new enrollments could not be made at the Turkish school in 2016.

## Conclusion

The results indicated that the MHC-SF and CLES+T scores obtained by the Portuguese Nursing students were higher than those of their Turkish and Lithuanian counterparts. It was also revealed that the students’ perceptions on the learning environment were affected by age; that supervision and teacher were affected by gender; and that learning environment, supervision and teacher were also affected by mental health. These findings show that the clinical learning environment has a strong effect on the Nursing students’ mental health. 

It is thought that the differences between the students belonging to three different cultures and who are candidates for the same profession are affected by the social structure and by individual and belief-related characteristics. In line with these results, it is important for nurse educators to recognize the unique needs of Nursing students to improve their students’ mental health during their clinical education.
